# Microencapsulation by Complex Coacervation of Lavender Oil Obtained by Steam Distillation at Semi-Industrial Scale

**DOI:** 10.3390/foods13182935

**Published:** 2024-09-17

**Authors:** István Székely-Szentmiklósi, Emőke Margit Rédai, Zoltán-István Szabó, Béla Kovács, Csilla Albert, Attila-Levente Gergely, Blanka Székely-Szentmiklósi, Emese Sipos

**Affiliations:** 1 Department of Industrial Pharmacy and Pharmaceutical Management, Specialty Pharmaceutical Sciences, “George Emil Palade" University of Medicine, Pharmacy, Science and Technology of Târgu Mureș, 540142 Târgu Mureș, Romania; istvan.szekely-szentmiklosi@umfst.ro (I.S.-S.); zoltan.szabo@umfst.ro (Z.-I.S.); emese.sipos@umfst.ro (E.S.); 2 Department of Pharmaceutical Technology and Cosmetology, Specialty Pharmaceutical Sciences, “George Emil Palade” University of Medicine, Pharmacy, Science and Technology of Târgu Mureș, 540142 Târgu Mureș, Romania; emoke.redai@umfst.ro; 3 Department of Biochemistry and the Chemistry of Environmental Factors, Fundamental Pharmaceutical Sciences, “George Emil Palade” University of Medicine, Pharmacy, Science and Technology of Târgu Mureș, 540142 Târgu Mureș, Romania; bela.kovacs@umfst.ro; 4 Department of Food Science, Sapientia Hungarian University of Transylvania, 530104 Miercurea Ciuc, Romania; albertcsilla@uni.sapientia.ro; 5 Department of Mechanical Engineering, Sapientia Hungarian University of Transylvania, 547367 Târgu Mures, Romania; agergely@ms.sapientia.ro; 6 Department of Pharmaceutical Chemistry, Specialty Pharmaceutical Sciences, “George Emil Palade” University of Medicine, Pharmacy, Science and Technology of Târgu Mureș, 540142 Târgu Mureș, Romania

**Keywords:** *Lavandula angustifolia* Mill., microencapsulation, complex coacervation

## Abstract

Lavender oil (LEO) is one of the most well-known essential oils worldwide which, besides its extensive application in aromatherapy, serves as raw material for various fields, including the food, cosmetic, and pharmaceutical industries. Accordingly, several global requirements were established to warrant its quality. Microencapsulation represents an emerging technology widely applied for the preservation of essential oils, simultaneously providing new ways of application. In the current study, lavender oil was obtained from the flowering tops of *Lavandula angustifolia* Mill. on a semi-industrial-scale steam distillation system. According to the GC-MS investigation, lavender oil obtained in the third year of cultivation met the *European Pharmacopoeia* standards for linalyl acetate and linalool contents ≈38% and ≈26%, respectively. Microcapsules (MCs) containing the so-obtained essential oil were successfully produced by complex coacervation technology between gum arabic (GA) and three different grades of type-A gelatin (GE). Optical microscopic investigations revealed a significant difference in particle size depending on the gelatin grade used. The variation observed for coacervates was well reflected on the scanning electron micrographs of the freeze-dried form. The highest encapsulation efficiency values were obtained by UV-VIS spectrophotometry for microcapsules produced using gelatin with the medium gel strength. FT-IR spectra confirmed the structural modifications attributed to microencapsulation. According to the GC-MS analysis of the freeze-dried form, the characteristic components of lavender oil were present in the composition of the encapsulated essential oil.

## 1. Introduction

Lavender oil (LEO) is perhaps the most widely known and used essential oil. It is included in the composition of many household and personal health-care products, and it is extensively used by the cosmetic industry, but its application in the food and pharmaceutic sectors is also significant [1].

*L. angustifolia* Mill., *L. stoechas* L., *L. latifolia*, and *L. X intermedia* are the four *Lavandula* species that are most widely cultivated for essential oil production. The most appreciated oil is provided by *Lavandula angustifolia* Mill. (LA), known as English lavender, formerly *Lavandula vera* or *Lavandula officinalis* [2]. The other three species give much higher yields at the expense of quality [3].

In different geographical regions, different cultivars of LA were developed in order to adapt the plant to specific climatic conditions and to increase the yield and/or to enhance essential oil quality. Some of the most popular cultivars of LA are Maillette, Hidcote, and Munstead [4]. Other factors that influence the qualitative and quantitative composition of the essential oil are genetic makeup, climate, reproduction, morphology variety, harvest time, soil pH, nutrient availability, and weather conditions. Moreover, the extraction method and distillation time might also have an impact on essential oil composition [5]. 

**Figure 1:**
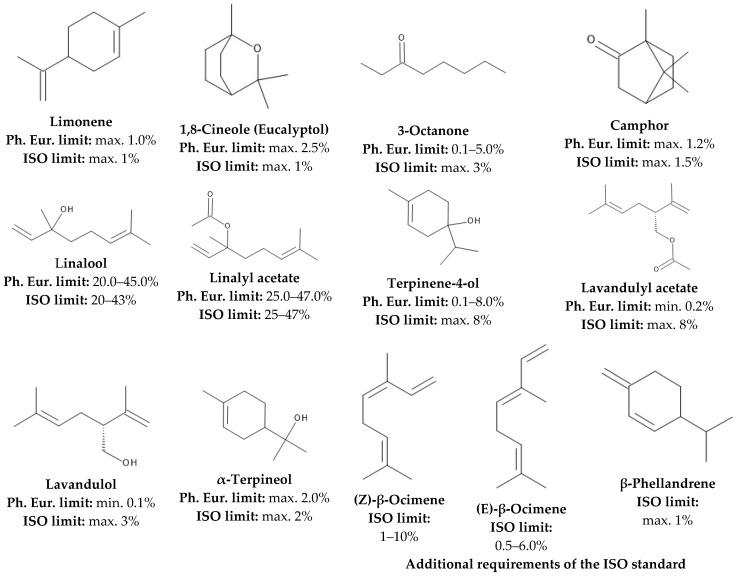
Requirements for the quantitative composition of LEO according to the *European Pharmacopoeia 11th Edition* and ISO 3515:2002 standard (“other origins” category).

One of the most exigent quality requirements is set for its application in the pharmaceutical field. It is officially listed in the *European Pharmacopoeia 11th Edition* (Ph. Eur.) under the name of lavender oil (lat. *Lavandulae aetheroleum*). The monograph establishes requirements for both the quality of the raw material and the method of production. It is prescribed that the flowering tops of LA (*Lavandula officinalis* Chaix.) have to be used for steam distillation [6]. Regarding its composition, the pharmacopeia establishes acceptance limits for ten components. It should be mentioned that the *European Pharmacopoeia* clearly distinguishes lavender oil from Spike lavender oil, which is described in a separate monograph and is defined as essential oil obtained via steam distillation of the flowering tops of Lavanadula latifolia Medik [7]. The lavender flower (lat. *Lavandulae flos*) pharmacopeial monograph specifies that the minimum essential oil (relative to the anhydrous drug) content has to be 13 mL/kg [8]. Another major quality-requirement standard for lavender oil is the ISO 3515:2002 (International Organization for Standardization), which contains acceptance criteria for three additional components in comparison to the *European Pharmacopoeia* and distinguishes lavender oil obtained from plants of different origins as follows: France—from seed; France—Maillette; Bulgaria; Russia; and other origins [2]. Figure 1 illustrates the relevant quality requirements regarding the composition of lavender oil according to the above-mentioned quality standards:

Quality standards are even more important since the essential oil of *Lavandula angustifolia* is one of the most frequently adulterated ones. In this regard, Wang et al., in their study involving the analyses of 72 essential oil samples by different analytical methods, including GC-MS, GC/Q-TOF, NMR, and chemometric analyses, described a high adulteration rate exceeding 25%. The possible addition of synthetic chemicals, poor quality, or adulteration was associated with high concentrations of terpinene-4-ol, cis/trans-β-ocimene, 3-octanone, camphor, or 1,8-cineole. A typical indicator of synthetic compound adulteration is the presence of trans-furano linalool oxide acetate [9]. The addition of the cheaper lavandin oil obtained from *Lavandula X intermedia* to lavender oil, which is one of the most common types of adulteration, gives the essential oil a sharper overtone due to the presence of camphor [10]. While there are areas of use where quality is not a primary consideration, in certain applications, it plays a central role. 

The use of microencapsulated lavender oil covers a wide range of industrial fields, including food applications, wound dressings, skincare textiles, eco-friendly weed, and pest management, fragrance-releasing textiles, and therapeutic applications. In active food packaging, microencapsulated LEO could make part of edible films and edible coatings or be present in the form of hydrogel beads. In these applications, its antibacterial properties can be exploited to prolong the shelf life of food products and to prevent microbial degradation of fruits and vegetables [11]. Along with other essential oils, lavender oil is listed as a GRAS (Generally Recognized as Safe) substance by the U.S. Food and Drug Administration (FDA) [12,13]. 

Pilicheva et al. applied emulsification and consequent spray drying method for the microencapsulation of lavender oil. The combination of gum arabic (GA) with maltodextrin in 3:1 ratio proved to be optimal concerning encapsulation efficiency and morphological characteristics. Researchers reported mean diameters between around 2.4 and 6.0 µm [14]. Valle et al. used complex coacervation between chitosan and gum arabic to encapsulate lavender oil for further textile functionalization. As emulsifier, Tween 20 was applied while as crosslinker tannic acid was used. The largest particle sizes observed by SEM on the textile substrate were 2 µm [15]. Xiao et al. optimized the pH, core/shell ratio, wall concentration, stirring speed, cross-linker type as well as homogenization speed applied during the complex coacervation between gelatin (GE) and gum arabic (GA). The drying process was performed by lyophilization. Diameter of particles was in the interval range of 41–196 µm [16]. 

The current study aimed to cultivate LA in our region; produce LEO by steam distillation at a semi-industrial scale; and transform it into solid form by complex coacervation, applying a GE-GA system using different GE grades, with glycerol as a crosslinker. It was also proposed to evaluate LEO quality in its free form vs. entrapped in freeze-dried microcapsules. 

## 2. Materials and Methods

### 2.1. Core Material 

**Figure 2:**
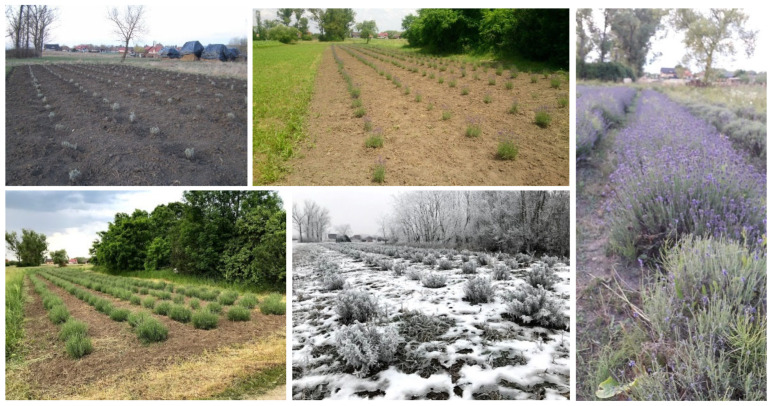
Different development stages of lavender plantation (*Lavandula angustifolia* Mill.).

Lavender oil was obtained via steam distillation from the flowering tops of LA cultivated in Romania, Transylvania Region, Mureș County, near the village of Dumbrăvioara, located in the floodplain of Mureș River, situated at 46°38′ latitude, 24°38′ longitude, and 336 m elevation. The plantation was established by planting seedlings (Figure 2) in 2019, and flowering tops were harvested in three consecutive years (2020, 2021, and 2022) and subjected to steam distillation. Lavender oil used in the current study for microencapsulation was obtained in 2022 and was stored at room temperature in airtight containers protected from light.

### 2.2. Shell Materials 

Gelatin type A of porcine origin with gel strength of 80–120 Bloom, approx. 175 Bloom, and approx. 300 Bloom values was purchased from Sigma Aldrich (St. Louis, MI, USA). Gum arabic from acacia tree was purchased from Sigma Aldrich Chemie (Steinheim, Germany). 

pH adjustment: For the pH adjustment, 0.5 N HCl acid was used. Hydrochloric acid was purchased from Silal Trading SRL (Bucharest, Romania). 

Crosslinker: Glycerol was purchased from Chimreactiv SRL (Bucharest, Romania).

### 2.3. Analytical Standards 

In GC-MS studies, linalyl acetate analytical standard (Sigma-Aldrich, Buchs, Switzerland) and linalool analytical standard (Sigma-Aldrich, Buchs, Switzerland) were used.

### 2.4. Steam Distillation 

**Figure 3:**
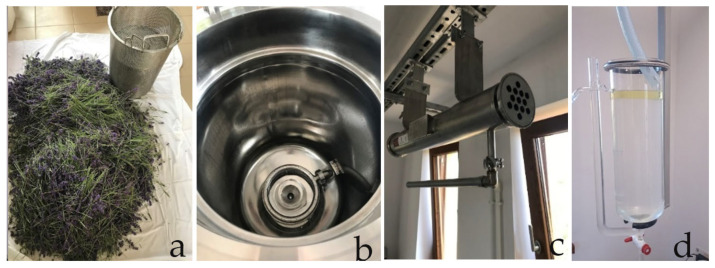
Parts of the semi-industrial-scale steam distillation system: perforated basket for biomaterial (**a**), bioreservoir with steam inlet ring (**b**), heat exchanger (**c**), and separation vessel (**d**).

Steam distillation of biomaterial was performed on a steam distillation system (Figure 3) consisting of a Kiskun Meridian stainless-steel bioreservoir of 50 L capacity (Kiskun Meridian, Kiskunfélegyhaza, Hungary), a Ghidini Maxi 16 type steam generator (Ghidini Benvenuto S.R.L., S. Giuliano Milanese, Italy), and an Aerre Inox DTS-type stainless-steel heat exchanger (Aerre Inox S.R.L., Fiesco Cremona, Italy), by applying a steam flow rate of approx. 8 L/h.

### 2.5. Microencapsulation Method 

Complex coacervation technology was applied according to the following major steps: 50 g of 3% *w*/*w* gum arabic aqueous solution was prepared by dissolving gum arabic at room temperature, under continuous stirring by the means of a magnetic stirrer (Arec, Velp Scientifica, Europe) at 800 rpm. Then, 50 g of 3% gelatin aqueous solution was prepared under continuous stirring and concomitant heating up to 50 ± 2 °C, using a magnetic stirrer, at 800 rpm. Three different gelatin type-A grades were used with gel-strength values as follows: approx. 175 Bloom (for experiment no. LA010), approx. 300 Bloom (for experiment no. LA011), and approx. 80–120 Bloom (for experiment no. LA013). In each case, 2.7 g of lavender essential oil was emulsified into the formerly prepared 3% *w*/*w* gelatin solution by using an IKA DI 18 B-type Ultra-Turrax (IKA^®^-Werke GmbH & Co. KG, Staufen, Germany) equipped with an IKA S 18 N-19 G-type dispersion tool (IKA^®^-Werke GmbH & Co. KG, Staufen, Germany) for 10 min, at a rotation speed of approx. 5000 rpm. This emulsion was added to the formerly prepared aqueous gum arabic solution, under continuous stirring, at approx. 50 °C. Then, the pH of the obtained mixture was adjusted to pH = 4.0 ± 0.05, using 0.5 N HCl solution. After cooling to 10 °C in an ice bath, the crosslinking agent, glycerol (1 g), was added to harden the formed shell. Separation and purification of microcapsules were performed by decantation and washing with purified water (volume 3 × 50 mL). After decantation, 50 mL of water was added to the microcapsule slurry, and the mixture was stirred by means of a magnetic stirrer for 20 min. The washing procedure was repeated three times. Simultaneously, microcapsules without essential oil, referred to as the placebo, were produced using the aforementioned method.

### 2.6. Freeze Drying 

**Figure 4:**
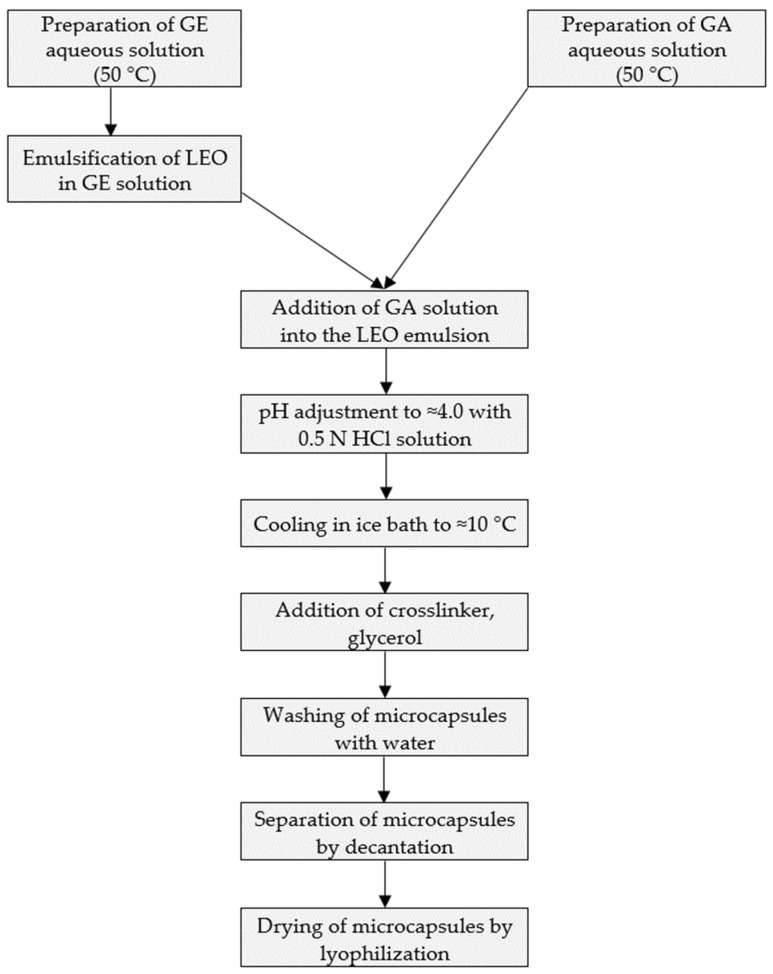
Process steps of LEO microcapsule formation.

Freeze drying of microcapsules was performed in a Biobase BK FD10S (Biobase, Ji-nan, China) equipment by applying 4 h of freezing at −55 °C, followed by 20 h of drying under a vacuum of 10 Pa. The technological process of microcapsule formation is presented in the following flowchart (Figure 4).

### 2.7. Optical Microscopy 

Optical microscopic investigation was performed on an Optika B-150D-BRPL (Optika SRL, Ponteranica, Italy) brightfield microscope equipped with an integrated 3.1 MP camera at magnifications of 40×, 100×, and 400×. For particle size measurement, Optika Proview 2020 software (Optika SRL, Ponteranica, Italy) was used. Microscopic specimens were prepared by bringing from the last washing cycle one droplet of microcapsule dispersion on a glass slide, and after doing so, a cover slip was gently applied. 

### 2.8. SEM Investigation

The morphology of the obtained products was investigated by scanning electron microscopic (SEM) imaging, with the use of a JEOL JSM-5200 scanning electron microscope (JEOL, Tokyo, Japan) at 10 kV potential. The samples were used as is (without sputter coating) and were fixed by conductive carbon adhesive tape.

### 2.9. FT-IR Spectroscopy

FT-IR spectroscopy of solid samples was performed on a Bruker Tensor 27 IR spectrophotometer (Bruker Optics, Ettlingen, Germany) controlled by the Opus software (version 7.2). IR spectra of the solid components, the physical mixture, and the prepared products were recorded using KBr (Sigma Aldrich, Merck, Darmstadt, Germany) pellets, in transmittance mode over 400–4000 cm^−1^ wavenumber range. The sample to KBr ratio was 1:100. Each sample was scanned 16 times, with a resolution of 2 cm^−1^. 

FT-IR spectroscopy of essential oil was performed on a Nicolet 380 IR spectrophotometer (Thermo Electron Corporation, Madison, WI, USA) controlled by the OMNIC 8.0 software.

### 2.10. UV-VIS Spectroscopic Analyses

UV-VIS spectroscopic determinations were performed on a Shimadzu UV-1800 spectrophotometer (Shimadzu Co., Kyoto, Japan), controlled by UVProbe 2.61 Software, using cuvettes made of special quartz glass with an optical path length of 10 mm (Hellma Analytics, Müllheim, Germany).

### 2.11. Essential Oil Content

LEO content in the microcapsules was determined by the UV-VIS spectroscopic method and quantified through the calculation of encapsulation efficiency and loading capacity. 

The amount of LEO in microcapsules was measured by performing the following steps: firstly, 100 mg of microcapsules was powdered in a mortar, 10 mL of absolute alcohol was added, and the mixture was magnetically stirred for 10 min to dissolve the total quantity of essential oil. Then, another 100 mg of microcapsules was weighed and stirred magnetically for 10 min, with 10 mL of absolute alcohol, and the supernatant was collected to recover the LEO remaining on the surface of the microcapsules. The alcoholic solutions were filtered, and after appropriate dilution, the 0.1 mg/mL microcapsule solutions were assayed spectrophotometrically at 233 nm. The LEO concentrations were calculated using a calibration curve built in the concentration range of 10–30 μg/mL. A similar method with the application of ethanol was applied by Gonçalves et al. [17].

The encapsulation efficiency (EE) refers to the amount of core material entrapped inside the microcapsules. This is an important index for evaluating the efficiency of the microencapsulation. It was calculated according to the following equation:

EE = m_m_/m_o_ · 100, (1)

where m_m_ is the amount (mg) of LEO contained in the microcapsules, and m_o_ is the initial LEO amount (mg) used. The amount of oil in microcapsules is determined by the difference between the total oil content (m_to_) and the surface oil content (m_so_):

m_m_ = m_to_ − m_so_, (2)

The loading capacity (LC) is defined as the amount of encapsulated core material relative to the total mass of the microcapsule and was calculated according to the following equation:

LC = m_m_/M · 100, (3)

where M is the total amount (mg) of microcapsules.

### 2.12. GC-MS Investigation

GC-MS analyses of the essential oil and microcapsules were performed using an Agilent single quadrupole mass spectrometer equipped with an inert mass selective detector (MSD-5977 A, Agilent Technologies, Santa Clara, CA, USA). This was directly connected to an Agilent 7890B gas chromatograph, which featured a split–splitless injector, a quick-swap assembly, an Agilent 7693 autosampler, and a HP-5MS fused silica capillary column (5% phenyl 95% dimethylpolysiloxane, 30 m × 0.25 mm internal diameter, and 0.25 μm film thickness, Agilent Technologies, USA). The HP-5MS column operated with an injector temperature of 250 °C. The oven temperature program began with an isothermal hold at 50 °C for 4 min, followed by a ramp of 4 °C/min to 220 °C, another isothermal hold for 2 min, a second ramp to 280 °C at 20 °C/min, and then a final isothermal hold for 15 min. 

Before the analysis, the essential oil was dissolved in hexane (1/100, *v*/*v*), and 1 μL of the solution was injected in 1:10 split mode. Extraction of essential oil from microcapsules was performed from 500 mg MC with 1 mL of hexane, followed by filtration. Helium was used as the carrier gas at a flow rate of 1 mL/min. GC-TIC profiles and mass spectra were obtained using the MSD ChemStation software, version F.01.01.2317 (Agilent). All mass spectra were acquired in the EI mode (scan range of *m*/*z* 45–600 and ionization energy of 70 eV). The electronic-impact ion source and MS quadrupole temperatures were set at 230 °C and 150 °C, respectively, with the MSD transfer line maintained at 280 °C. The NIST 14 MS spectrum library was used for spectrum searching and identification.

## 3. Results and Discussion

### 3.1. GC-MS Investigation of Essential Oil Composition

#### 3.1.1. Free Form of Essential Oil

The LEO was analyzed by GC-MS technique in 2022 following production by steam distillation and in 2024 after 2 years storage, for shelf-life evaluation of self-produced LEO. Table 1 contains the results in comparison to requirements of *European Pharmacopoeia 11th Edition* and ISO 3515:2002 (“other origins” category) standard.

According to the GC-MS analysis and identification of components performed by RT and NIST 14 spectral libraries the lavender oil obtained by steam distillation in 2022 in the third year of cultivation fulfilled the quality requirements of the *European Pharmacopoeia* regarding composition. The ISO requirements were also fulfilled, except for (Z)-β-ocimene and (E)-β-ocimene contents, which were present in very low amounts, below the lower limit of 1% and 0.5%, respectively, defined by the ISO standard. After 2 years of storage, the linalyl acetate and linalool contents decreased, with an amount of 7.23% in the case of linalyl acetate (from 38.38% to 31.15%) and with an amount of 7.51% in the case of linalool (from 26.23% to 18.72%), with the latter falling below the 20% lower limit of ISO and Ph. Eur. requirement. At the same time, the (E)-β-ocimene content exceeded the upper-limit value of the ISO standard reaching more than 9%. A possible cause of this increase might be the deacetylation of linalyl acetate, which might result in β-myrcene, cis β-ocimene, or trans-β-ocimene [18]. Another theoretically potential chemical pathway for the explanation of the trans-β-ocimene level’s increase during storage would involve the dehydration of linalool [19]. Furthermore, limonene content has to be mentioned, which was situated after 2 years slightly above the 1.0% limit. It should be emphasized that even after two years of storage, all the other components remained in the specified thresholds.

#### 3.1.2. Microencapsulated Form of Essential Oil

The compositions of LEO in its free form and within microcapsules prepared with the three different types of GE grades are presented in Table 2.

Although the major constituents of the essential oil remained the same following microencapsulation, it could be observed that plenty of compounds present in the composition of free essential oil were lost during the technological process. Thus, depending on the type of gelatin used, instead of the initial 38 components, only 10, 8, or 9 were present in the composition of microencapsulated essential oil. Especially compounds with high volatility lacking hydrophilic radicals and having small molecular size were lost following complex coacervation and freeze drying. In this regard, throughout the technological process, emulsification and freeze-drying steps might play a crucial role, as during these process steps, moderate heating (to 50 °C) and reduced pressure, respectively, are applied. 

Table 3 summarizes the fundamental properties of the compounds identified in the composition of microencapsulated essential oils.

In general, components present in concentrations below 1.5% in the essential oil could not be found in the composition of the encapsulated essential oil. The exception is menthol, which was eliminated despite being present at 2.89%. Caryophyllene oxide and tau cadinol could be mentioned as counterexamples which were present in the composition of LEO at concentrations of 0.52% and 1.13%, respectively, and were also detected in all three microcapsules. Monoterpene ketones were lost following the technological process. Except for cis- and trans-beta-ocimenes, monoterpenes lacking a functional group could not be found in the composition of microencapsulated LEO. Four out of six monoterpene alcohols were identified in one or more forms of MC, and two out of five monoterpene esters were detected. Among the sesquiterpenes, caryophyllene and beta-farnesene, as well as caryophyllene oxide (a sesquiterpene oxide) and tau cadinol (a sesquiterpene alcohol), were preserved.

An interesting phenomenon was noticed in relation to the linalyl acetate content. In each microencapsulation experiment, an accumulation of linalyl acetate was observed, reaching values in the range of 54–61%, compared to 31%, as it was present in the composition of free LEO. Presumably, hydrophobic and/or electrostatic interactions between linalyl acetate and the microcapsule shell lie at the origin of this preferential entrapment. The optimization of process parameters will be required to elucidate the major factors affecting the essential oil composition. As the volatility of constituents might play an important role in this regard, process optimization has to be focused on temperature during the emulsification of essential oil as well as the extent of the applied vacuum during freeze drying.

In comparison to the composition of free essential oil, a new component was identified in the microencapsulated form. Geranyl acetate appears in the composition of encapsulated essential oil in the case of each experiment, at levels between 1.16 and 1.41%. Presumably, this might derive from the allylic rearrangement of linalyl acetate [18]. 

### 3.2. Results of Optical Microscopic Investigation

**Figure 5:**
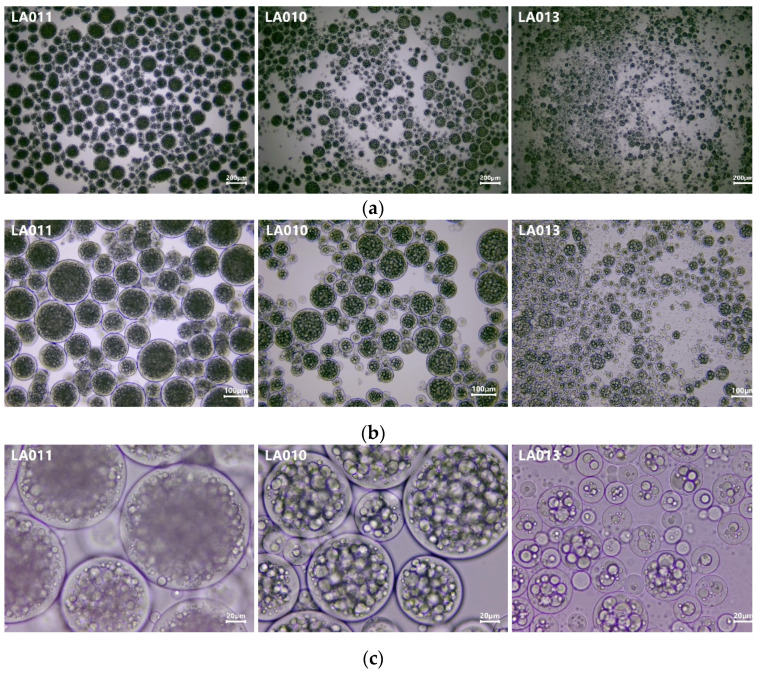
Optical microscopic images of experiments (LA011, LA010, and LA013) at different magnifications: (**a**) 40×, (**b**) 100×, and (**c**) 400×.

As presented in Figure 5, the optical microscopic images were captured at three different magnification levels.

The polydispersity of microcapsule dimensions is well reflected on images at 40× and 100× magnifications. A polynucleated structure is predominantly characteristic to experiments incorporating the medium- and high-gel-strength gelatin. In the case of experiment no. LA013, prepared with gelatin of 80–120 Bloom, the mono- and oligonucleated forms are the most prevalent.

The results of the particle size measurements are disclosed in Table 4.

The particle size of the formed microcapsules decreased with the gel-strength value of gelatin (Table 4). The difference observed in the particle size of microcapsules formed is in good agreement with the research work of Peters et al., who described the increase in the diameter of microcapsules with the increase in Bloom grade for type-A gelatins. [23] The viscosity of gelatin solutions might be one of the most important factors which stay at the background of this phenomenon. Reduced shear forces due to the higher viscosity facilitate the formation of larger microcapsules. As different applications might require tailored particle sizes of microcapsules, this approach of modulating the microcapsule dimension represents one of the viable solutions for achieving a specific microcapsule size. The impact of other formulation factors (e.g., core/shell ratio, shell composition, and crosslinker type) and technological parameters related to viscosity and/or shear forces (temperature of solutions prepared and stirring rate during coacervate formation) requires a thorough investigation and systematic assessment. Another possibility to fine-tune the particle size of microcapsules might be accomplished by the combination of gelatin types at different proportions. 

### 3.3. Morphological Investigation of Freeze-Dried Form

#### 3.3.1. Macroscopic Aspect

**Figure 6:**
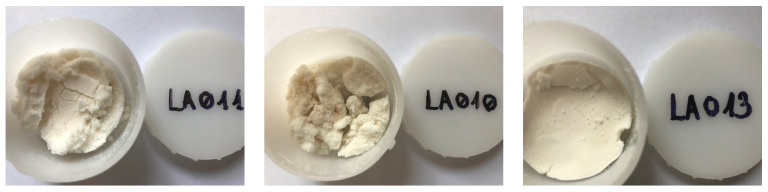
Macroscopic aspect of the lyophilized samples obtained with different gelatin grades (exp. no. LA011—300 Bloom; exp. no. LA010—175 Bloom; and exp. no. LA013—80–120 Bloom).

Following lyophilization, the samples presented different macroscopic aspects. While freeze-dried forms obtained with gelatin having the highest and medium gel strength (experiment no. LA011 and LA010) exhibited a granular structure, the application of gelatin having 80–120 Bloom conferred a more compact structure with a smoother surface (Figure 6).

#### 3.3.2. Electronmicroscopic Investigation

**Figure 7:**
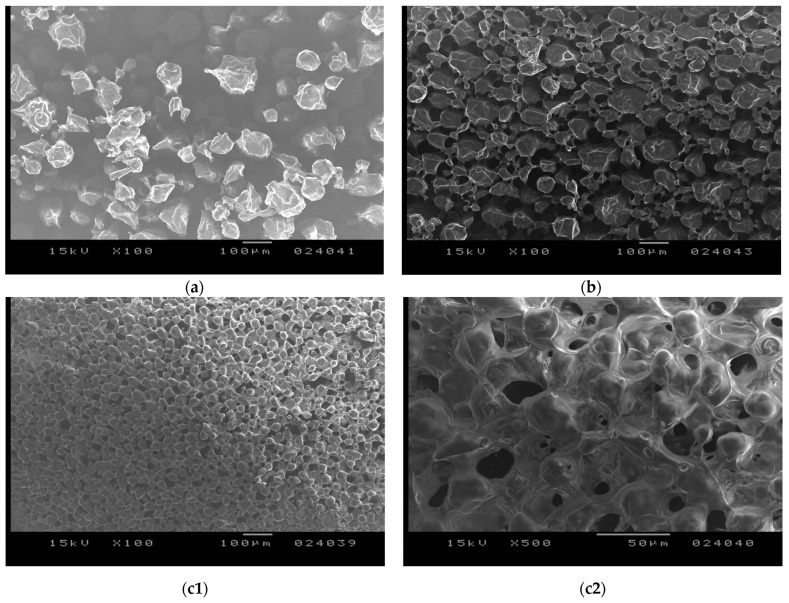
SEM image of freeze-dried microcapsules prepared with different gelatin grades: (**a**) experiment no. LA011, manufactured with gelatin, approx. 300 Bloom—100×; (**b**) experiment no. LA010, manufactured with gelatin, approx. 175 Bloom—100×; and (**c1**) experiment no. LA013, manufactured with gelatin, approx. 80–120 Bloom—100× and (**c2**) 500× magnification.

On the electronmicrographs (Figure 7), in the case of each experiment, embedded globular structures could be observed as interconnected with each other. The net-like structure of the freeze-dried form observed on the SEM images characterized by interconnected lyophilized particles could be attributed to the formation of solid bridges.

This type of structure is a particularity of freeze-dried microcapsules and was reported by several authors [24,25,26]. Unlike other drying methods, which imply the dynamic effect of an air stream (e.g., spray drying), facilitating the separation of microcapsules during the process, in the case of freeze-drying, microcapsules remain stuck together to some extent in a net-like structure through the formation of solid bridges. According to our experience, from the freeze-dried form to the addition of water, the microcapsules in their original form could be easily obtained. Rehydration occurs in a very rapid manner, along with the formation of microcapsule dispersion. Thus, the lyophilized form would be appropriate for the long-term storage of microcapsules in solid state, and before utilization, rehydration could be performed with the addition of water. Nevertheless, if microcapsules are intended to be used in dry form, a material with better rheological properties might be required. In this concern, the application of another drying method (e.g., spray drying) could represent a solution that can be assumed to be the consequence of the type of drying process applied. 

The SEM images, besides revealing the intrinsic structure of the freeze-dried form, confirmed the variation noticed in particle size by optical microscopy and also the difference in the compactness of freeze-dried forms observed macroscopically. In the case of gelatin with the highest Bloom strength (exp. no. LA011), almost entirely separated particles could be observed, while when gelatin with a lower Bloom value was applied, a more compact structure was formed.

### 3.4. Determination of Encapsulation Efficiency and Loading Capacity by UV Spectroscopy

To determine the encapsulation efficiency, UV–visible spectroscopic measurements were conducted. Initially, the UV-VIS spectrum of the crude essential oil was recorded at appropriate dilution, revealing a well-distinguished absorption peak at 233 nm. Subsequent measurements were performed at this wavelength, and concentrations were determined using a calibration curve. 

The calibration diagram was constructed using the concentrations and measured absorbances of seven different alcoholic solutions of LEO, ranging from 10 to 30 μg/mL. The resulting equation, y = 0.0175x − 0.0101 with R^2^ = 0.993, demonstrates a high linear correlation between LEO concentrations and absorbances at 233 nm.

The absorbances of the synthesized microcapsules dissolved in ethanol were also measured. The LEO-containing microcapsules presented a maximum absorbance at 233 nm, whereas the microcapsules without oil (PL) showed no absorption maximum, indicating that the presence of oil can be detected in the microcapsule solutions. 

The results obtained for the encapsulation efficiency (EE) and loading capacity (LC) are presented in Table 5.

While in the case of gelatin type-A grades with intermediate and high gel strengths, high EE values were recorded (≈92% for intermediate bloom strength respective ≈81% for highest bloom strength), in the case of experiment no. LA013, where gelatin with the lowest gel strength was used, the encapsulation efficiency reached only ≈56%, revealing a substantial loss in the essential oil content during the technological process. This phenomenon might be attributed to the smaller particle size of the formed microcapsules obtained and the resulting higher specific surface area, which makes microcapsules more prone to essential oil loss during the technological process following coacervate formation. In this regard, the technological parameters of the freeze-drying step might play a critical role, which requires further optimization. Xiao et al., in their study on complex coacervation of LEO under different formulation and technological parameters between gum arabic and gelatin type B, reported encapsulation-efficiency values between ≈14% and 66%. Determination was performed by the UV-VIS spectrophotometric method. The authors hypothesized that some losses might occur due to volatilization in the course of the emulsification and coacervation when a temperature of approx. 50 °C was used [16]. Ocak applied the same analytical technique for EE measurement and used 0.3% Tween solution for the extraction of LEO from microcapsules obtained via complex coacervation between collagen hydrolysate extracted from solid wastes and chitosan. EE values between 36.8 and 60.1% were reported [27]. Burhan et al. also applied the UV-VIS spectrophotometric method and used it for the extraction of LEO 5 *w*/*v*% sodium lauryl sulfate solution. Microencapsulation was performed via spray drying, and shell material GA and maltodextrin were applied. A maximum EE value of 77.9% was obtained [28]. In the framework of a full factorial experimental design, Rungwasantisuk and Raibhu, for complex coacervate of lavender oil by using type-A gelatin with 170–175 Bloom, gum arabicum, and glutaraldehyde as crosslinker, along with freeze drying, obtained EE values in the range of ≈67–85% [29]. Zhang et al. tested different coating materials for the microencapsulation of LEO. The presence of gelatin besides GA and sodium caseinate improved volatile retention, as EE increased dramatically from 28.6% to 65.9% [30]. 

### 3.5. Results of FT-IR Spectroscopic Investigations

**Figure 8:**
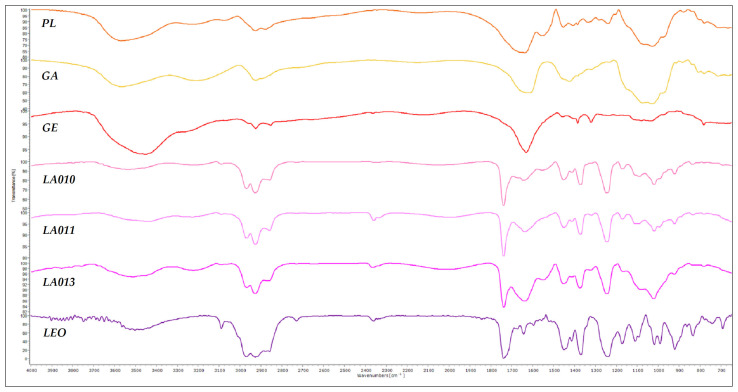
The FT-IR spectra of oil-free microcapsule (PL), gelatin (GE), gum arabic (GA), microcapsules prepared with three different gelatin grades (LA010, LA011, and LA013), and essential oil (LEO).

The spectrum of the oil-free microcapsule was compared with the spectra of its components and with those of microcapsules produced using three different types of gelatin, while also considering the spectrum of the pure essential oil in the prior analysis (Figure 8).

Gum arabic is a complex mixture of polysaccharides and glycoproteins [31]. Its FT-IR spectrum (Figure 8) is characterized by a strong absorption band at 3567 cm^−1^, attributed to the -OH stretching vibration of free or hydrogen-bonded hydroxyl groups associated with the polysaccharide structure. The peak at 3212 cm^−1^ corresponds to the stretching vibrations of N-H bonds, likely due to the glycoprotein part of GA structure [32]. A weaker peak at 2925 cm^−1^, characteristic of C-H stretching vibrations in alkyl groups, suggests the presence of hydrocarbon chains within the polysaccharide structure. Additionally, peaks at 1617 cm^−1^ and 1425 cm^−1^ represent asymmetrical and symmetrical stretching vibrations of the carboxylate groups (-COO^−^) [28,33] primarily attributed to glucuronic acid units. The strong absorption band at 1031 cm^−1^ corresponds to asymmetric stretching vibrations of -C-O-C and -C-O, which are common in polysaccharides, indicating glycosidic linkages and hydroxyl groups in arabic gum [34,35].

Gelatin is a polypeptide derived from collagen, primarily composed of the amino acids glycine, proline, hydroxyproline, and glutamic acid [36,37]. The FT-IR spectrum of gelatin displays a broad peak at 3453 cm^−1^ and a smaller one at 3225 cm^−1^, which reflect contributions from functional groups such as the N-H stretching of amide bonds and -NH_2_ groups, as well as O-H stretching vibrations from hydroxyl groups in amino acid side chains [38,39]. The peak at 2924 cm^−1^ arises from the stretching vibrations of C-H bonds in the aliphatic chains of amino acid residues, while the peak at 1385 cm^−1^ is associated with the bending vibrations of C-H bonds in methyl groups, indicating amino acids with methyl-containing side chains. The prominent peak at 1632 cm^−1^ is primarily due to the C=O stretching vibrations of the amide groups in the protein backbone, characteristic of proteins and polypeptides reflecting the peptide bond structure. Lastly, the peak at 1032 cm^−1^ can be attributed to the C-N stretching vibrations of amine groups or the C-O stretching of hydroxyl groups, indicative of the complex structure of amino acid residues and peptide bonds in gelatin [32,34]. 

During complex coacervation, the free negatively charged carboxylic groups of polysaccharides interact with the positively charged amino groups of proteins to form complexes. Figure 8 shows the FT-IR spectrum of the blank coacervates (PL), including characteristic peaks of both GA and GE at 3570 cm^−1^, 2225 cm^−1^, 2926 cm^−1^, and 1638 cm^−1^, while the characteristic peak of the LEO at 1425 cm^−1^ is missing. Similar findings have been reported by other authors for GA-GE coacervates, attributing this to the involvement of GA’s carboxylic groups in electrostatic interactions with the amino groups of the protein. The appearance of new peaks at 1240 cm^−1^ and 1029 cm^−1^ indicates the presence of N-H groups that can form hydrogen bonds and participate in electrostatic interactions when protonated (NH_3_^+^), as well as C-O stretching in arabic gum, which supports the presence of carboxylate groups (COO^−^) contributing to negative charges [32].

The peak observed in the FT-IR spectrum of LEO at 3486 cm^−1^ indicates O-H stretching vibrations, which are characteristic of hydroxyl groups commonly found in alcohols and phenols. In lavender oil, this could be due to the presence of compounds like linalool. The peak at wavenumber 2930 cm^−1^ is associated with C-H stretching vibrations in aliphatic hydrocarbons, reflecting the presence of methylene (-CH_2_^−^) and methyl (-CH_3_) groups in the diverse terpenes, and terpenoids of lavender oil. The intense band at 1740 cm^−1^ is indicative of carbonyl (C=O) stretching vibrations, characteristic of ester, aldehyde, and ketone functional groups, often corresponding to the ester functional group in compounds such as linalyl acetate [28]. The peak at 1455 cm^−1^ is due to C-H bending (scissoring) vibrations in -CH_2_ and -CH_3_ groups, while the one at 1374 cm^−1^ is related to C-H bending (wagging) vibrations in methyl (-CH_3_) groups. The peak at 1244 cm^−1^ can be attributed to C-O stretching vibrations in esters and ethers; it aligns with the presence of ester groups in components like linalyl acetate. The peak at 1021 cm^−1^ is due to C-O stretching vibration in alcohols, ethers, and esters, like linalool and linalyl acetate. The peak at 924 cm^−1^ could be associated with out-of-plane bending vibrations of C-H bonds, suggesting the presence of unsaturated compounds in lavender oil [28,34,35]. 

To investigate the intermolecular interactions between the wall materials and the core material, the FT-IR spectra of the LEO-loaded microcapsules were compared with the spectrum of essential oil (Figure 8 LEO) and the spectrum of the blank microcapsules (Figure 8 PL), as well. In all three oil-containing microcapsules, the characteristic peaks of LEO at 2930 cm^−1^, 1740 cm^−1^, 1455 cm^−1^, 1374 cm^−1^, and 1020 cm^−1^ can be observed. There is also an overlap of some peaks of PL with those of LEO in the wavelength region of 3450–3600 cm^−1^, as well as a characteristic peak of PL at 3225 cm^−1^. 

According to Ayah M. Burhan et al., the preservation of the LEO peaks within the microparticle matrix indicates the stability and structural preservation of the encapsulated lavender oil [28]. Zhang et al. stated that the fact the position of these peaks remained almost unchanged and no new chemical bonds were found in spectra of microcapsules suggested that the essential oil was successfully encapsulated and there was no significant interaction between essential oil and wall materials [30]. Xiao et al. also noted that the absence of new peaks, when compared to the spectra of PL and LEO, indicates that no new chemical bonds were formed. This further confirms the formation of complexes promoted by physical interaction, such as electrostatic interaction rather than chemical reactions, and that there was no significant interaction between the GE-GA complex and lavender oil [16]. 

## 4. Conclusions

This study investigated the composition of lavender essential oil (LEO) obtained via steam distillation from *Lavandula angustifolia* Mill., cultivated in Romania, Transylvania Region, and its microencapsulation using complex coacervation. The LEO produced met the *European Pharmacopoeia* standards for key components such as linalool and linalyl acetate. After two years of storage, however, a decrease in linalool and linalyl acetate concentrations was observed, suggesting chemical changes in the oil during storage. The microencapsulation of LEO was successfully achieved using complex coacervation between gum arabic and three different grades of gelatin type A, with glycerol as the crosslinking agent. The process resulted in microcapsules with varying particle sizes depending on the gelatin grade used. The encapsulation efficiency (EE) was highest with gelatin of medium gel strength, demonstrating the method’s effectiveness for entrapping LEO. The FT-IR analysis confirmed that the shell structure formed through electrostatic interactions between GA and GE and showed no significant chemical interactions between the shell material and the core, preserving the integrity of the essential oil. The study found that linalyl acetate concentrations increased in the encapsulated form, indicating preferential entrapment. This finding suggests that while microencapsulation helps retain key components of the essential oil, some volatile compounds may be lost during the process. The results contribute to the field by demonstrating that microencapsulation using different gelatin grades allows for the modulation of particle size, a critical factor in applications where microcapsule dimensions are essential. Future work should focus on optimizing the process to improve the retention of more volatile components and assessing the stability of encapsulated lavender oil for industrial applications.

## Figures and Tables

**Table 1:** Controlled components of lavender oil according to *European Pharmacopoeia 11th Edition* and ISO 3515:2002 (“other origins” category).

Component	ISO Requirement (Other Origins)	Ph. Eur. Requirement	Lavender Oil Obtained by Steam Distillation in 2022	
			Initial	After 2 Years of Storage
	%		Peak, %	
Linalyl acetate *	25–47	25.0–47.0	38.38	31.15
Linalool *	20–43	20.0–45.0	26.23	18.72
(Z)-β-ocimene **	1–10	N/A	0.07	N/A
Lavandulyl acetate	≤8	≥0.2	3.54	2.57
Terpinen-4-ol	≤8	0.1–8.0	7.38	5.58
(E)-β-ocimene **	0.5–6.0	N/A	0.09	9.24
3-Octanone	≤3	0.1–5.0	0.38	0.41
1,8-Cineole	≤3	≤2.5	0.26	0.50
Lavandulol	≤3	≥ 0.1	0.39	0.18
α-terpineol	≤2	≤2.0	1.16	1.07
Camphor	≤1.5	≤1.2	0.57	0.27
Limonene	≤1	≤1.0	0.19	1.10
β-phellandrene **	≤1	N/A	0.06	N/A

* GC-MS identification was confirmed by analytical standards besides RT and NIST 14 spectral libraries. ** Components controlled additionally by the ISO standard.

**Table 2:** GC-MS results of controlled components by ISO for free and microencapsulated LEO (exp. no. LA010—produced with GE, approx. 175 Bloom; exp. no. LA011—produced with GE, approx. 300 Bloom; and exp. no. LA013—produced with GE, 80–120 Bloom).

No.	Compound *	Retention Time (min)	LEO	LA010	LA011	LA013
			Peak, %			
1	Thujene	5.225	0.32	-	-	-
2	Alpha-pinene	5.452	0.53	-	-	-
3	Camphene	5.942	0.40	-	-	-
4	Beta-pinene	6.951	0.30	-	-	-
5	3-Octanone	7.287	0.41	-	-	-
6	Beta-myrcene	7.507	1.18	-	-	-
7	3-Carene	8.610	0.23	-	-	-
8	P-Cymene	9.259	0.49	-	-	-
9	Limonene	9.507	1.10	-	-	-
10	1,8 Cineole	9.658	0.50	-	-	-
11	Trans-beta-ocimene	10.155	9.24	6.31	-	2.39
12	Beta-ocimene	10.769	2.53	1.50	-	-
13	Gamma-terpinene	11.369	0.43	-	-	-
14	Terpinolene	13.080	0.30	-	-	-
15	Linalool	13.906	18.72	18.60	22.01	19.67
16	Neo-allo-ocimene	14.506	0.39	-	-	-
17	Camphor	15.306	0.27	-	-	-
18	1-Menthone	16.464	1.45	-	-	-
19	Endo-borneol	17.002	1.58	-	1.57	-
20	Lavandulol	17.193	0.18	-	-	-
21	Menthol	17.395	2.89	-	-	-
22	Terpinen-4-ol	17.623	5.58	3.38	3.17	3.16
23	Cryptone	17.983	0.16	-	-	-
24	Terpineol	18.209	1.07	-	1.37	
25	Isobornyl acetate	18.395	0.22	-	-	
26	Linalyl acetate	21.243	31.15	55.82	54.27	60.94
27	Bornyl acetate	22.255	0.17	-	-	-
28	Lavandulyl acetate	22.511	2.57	3.01	3.11	3.31
29	Neryl acetate	25.187	0.76	-	-	-
30	Geranyl acetate	25.856	N/A	1.35	1.16	1.41
31	Beta-bourbonene	25.862	1.21	-	-	-
32	Caryophyllene	27.069	4.67	3.80	-	2.63
33	Trans-alpha-bergamotene	27.604	0.21	-	-	-
34	Humulene	28.183	0.10	-	-	-
35	Beta-farnesene	28.324	4.35	3.41	-	2.20
36	Germacrene D	29.096	0.33	-	-	-
37	Gamma-cadinene	30.131	0.96	-	-	-
38	Caryophyllene oxide	32.234/32.234	0.52	1.31	4.52	2.35
39	Tau cadinol	33.902	1.13	1.51	1.48	1.93
	Total identified		98.6	100	92.66	99.99

* In the order of retention times.

**Table 3:** Fundamental characteristics of LEO components.

Compound	Molecular Weight	Boiling Point * (°C)	Group of Compounds	Terpene Category
Thujene	136.23	150–152	Alkene	Monoterpene
Alpha-pinene	136.23	155–157	Alkene	Monoterpene
Camphene	136.23	156–160	Alkene	Monoterpene
Beta-pinene	136.23	165–166	Alkene	Monoterpene
3-Octanone	128.21	167–170	Ketone	Ketone (not a terpene)
Beta-myrcene	136.23	166–167	Alkene	Monoterpene
3-Carene	136.23	169–174	Alkene	Monoterpene
P-cymene	134.22	176–178	Aromatic Hydrocarbon	Monoterpene
Limonene	136.23	175–176	Alkene	Monoterpene
1,8 Cineole	154.25	176–177	Ether	Monoterpene
Trans-beta-ocimene ^2^	136.23	174–175	Alkene	Monoterpene
Beta-ocimene ^1^	136.23	174–175	Alkene	Monoterpene
Gamma-terpinene	136.23	181–183	Alkene	Monoterpene
Terpinolene	136.23	183–185	Alkene	Monoterpene
Linalool ^3^	154.25	197–198	Alcohol	Monoterpene alcohol
Neo-allo-ocimene	136.23	188–189 (predicted)	Alkene	Monoterpene
Camphor	152.23	204–209	Ketone	Monoterpene ketone
1-Menthone	154.25	207–210	Ketone	Monoterpene ketone
Endo-borneol ^1^	154.25	210–215	Alcohol	Monoterpene alcohol
Lavandulol	154.25	229–230	Alcohol	Monoterpene alcohol
Menthol	156.26	214–216	Alcohol	Monoterpene alcohol
Terpinen-4-ol ^3^	154.25	209–212	Alcohol	Monoterpene alcohol
Cryptone	138.21	198 (experim.) 208 (predicted)	Ketone	Monoterpene ketone
Terpineol ^1^	154.25	218–221	Alcohol	Monoterpene alcohol
Isobornyl acetate	196.29	220–227	Ester	Monoterpene ester
Linalyl acetate ^3^	196.29	220–221	Ester	Monoterpene ester
Bornyl acetate	196.29	220–227	Ester	Monoterpene ester
Lavandulyl acetate ^3^	196.29	228.7 ± 19.0 (predicted)	Ester	Monoterpene ester
Neryl acetate	196.29	262–265	Ester	Monoterpene ester
Beta-bourbonene	204.35	255.9 ± 7.0 (predicted)	Alkene	Sesquiterpene
Caryophyllene ^2^	204.35	256–259	Alkene	Sesquiterpene
Trans-alpha-bergamotene	204.35	259–260	Alkene	Sesquiterpene
Humulene	204.35	166–168	Alkene	Sesquiterpene
Beta-farnesene ^2^	204.35	206	Alkene	Sesquiterpene
Germacrene D	204.35	279.7 ± 20.0 (predicted)	alkene	Sesquiterpene
Gamma-cadinene	204.35	271–276	alkene	Sesquiterpene
Caryophyllene oxide ^3^	220.35	279.7 ± 19.0 (predicted)	Epoxide	Sesquiterpene oxide
Tau cadinol ^3^	222.37	303.4 ± 31.0 (predicted)	Alcohol	Sesquiterpene alcohol

* Data according to References [20,21,22]. ^1^ Component preserved in 1 out of 3 microencapsulation experiments. ^2^ Component preserved in 2 out of 3 microencapsulation experiments. ^3^ Component preserved in 3 out of 3 microencapsulation experiments.

**Table 4:** Particle size characteristics of microcapsules (MCs) containing LEO manufactured with different gelatin grades.

	Particle Size (µm)			SD	RSD%	Median (µm)	Polydispersity Index
Exp. No.	Average	Min	Max				
LA011	101 ^1^	25	273.5	43.24	42.69	98.1	0.18
LA010	71 ^2^	25.8	197.6	32.17	45.20	62.6	0.20
LA013	31 ^3^	8.5	100.7	13.15	42.94	28.3	0.18

^1^ n = 455; ^2^ n = 631; ^3^ n = 627.

**Table 5:** Results of encapsulation efficiency and loading capacity.

Exp. No.	LEO in Microcapsules	LEO Concentration mg/mL	Concentration of MC Solution mg/mL	Theoretical Oil Content mg/mL MC	EE%	LC%
LA011	Total oil	4.00	10	4.03	91.71	36.96
	Surface oil	0.31				
LA010	Total oil	3.49	10	4.03	81.46	32.83
	Surface oil	0.21				
LA013	Total oil	2.79	10	4.03	56.36	22.71
	Surface oil	0.52				

## Data Availability

The original contributions presented in the study are included in the article; further inquiries can be directed to the corresponding author.

## References

[1] Wells R., Truong F., Adal A.M., Sarker L.S., Mahmoud S.S. (2018). Lavandula Essential Oils: A Current Review of Applications in Medicinal, Food, and Cosmetic Industries of Lavender. Nat. Prod. Commun..

[2] Pokajewicz K., Białoń M., Svydenko L., Fedin R., Hudz N. (2021). Chemical Composition of the Essential Oil of the New Cultivars of Lavandula Angustifolia Mill. Bred in Ukraine. Molecules.

[3] Kozuharova E., Simeonov V., Batovska D., Stoycheva C., Valchev H., Benbassat N. (2023). Chemical Composition and Comparative Analysis of Lavender Essential Oil Samples from Bulgaria in Relation to the Pharmacological Effects. Pharmacia.

[4] Crișan I., Ona A., Vârban D., Muntean L., Vârban R., Stoie A., Mihăiescu T., Morea A. (2023). Current Trends for Lavender (*Lavandula angustifolia* Mill.) Crops and Products with Emphasis on Essential Oil Quality. Plants.

[5] Rathore S., Kumar R. (2022). Essential Oil Content and Compositional Variability of Lavandula Species Cultivated in the Mid Hill Conditions of the Western Himalaya. Molecules.

[6] European Medicines Agency (EMA) (2022). Community Herbal Monograph on Lavender Oil (04/2023:2972). European Pharmacopoeia (Ph. Eur.).

[7] European Medicines Agency (EMA) (2022). Community Herbal Monograph on Spike Lavender Oil (07/2018: 2419). European Pharmacopoeia (Ph. Eur.).

[8] European Medicines Agency (EMA) (2022). Community Herbal Monograph on Lavender Flower (07/2018:1534). European Pharmacopoeia (Ph. Eur.).

[9] Wang M., Zhao J., Ali Z., Avonto C., Khan I.A. (2021). A Novel Approach for Lavender Essential Oil Authentication and Quality Assessment. J. Pharm. Biomed. Anal..

[10] Beale D.J., Morrison P.D., Karpe A.V., Dunn M.S. (2017). Chemometric Analysis of Lavender Essential Oils Using Targeted and Untargeted GC-MS Acquired Data for the Rapid Identification and Characterization of Oil Quality. Molecules.

[11] Hedayati S., Tarahi M., Iraji A., Hashempur M.H. (2024). Recent Developments in the Encapsulation of Lavender Essential Oil. Adv. Colloid Interface Sci..

[12] Jackson-Davis A., White S., Kassama L.S., Coleman S., Shaw A., Mendonca A., Cooper B., Thomas-Popo E., Gordon K., London L. (2023). A Review of Regulatory Standards and Advances in Essential Oils as Antimicrobials in Foods. J. Food Prot..

[13] Fukushima S., Cohen S.M., Eisenbrand G., Gooderham N.J., Guengerich F.P., Hecht S.S., Rietjens I.M.C.M., Rosol T.J., Davidsen J.M., Harman C.L. (2020). FEMA GRAS Assessment of Natural Flavor Complexes: Lavender, Guaiac Coriander-Derived and Related Flavoring Ingredients. Food Chem. Toxicol..

[14] Pilicheva B., Uzunova Y., Katsarov P. (2021). Comparative Study on Microencapsulation of Lavender (Lavandula Angustifolia Mill.) and Peppermint (*Mentha piperita* L.) Essential Oils via Spray-Drying Technique. Molecules.

[15] Valle R.d.C.S.C., Valle J.A.B., Bezerra F.M., Correia J., da Costa C., Martí M., Coderch L., López A., Arias M.J.L. (2023). Application of Lavender-Oil Microcapsules to Functionalized PET Fibers. Polymers.

[16] Xiao Z., Liu W., Zhu G., Zhou R., Niu Y. (2014). Production and Characterization of Multinuclear Microcapsules Encapsulating Lavender Oil by Complex Coacervation. Flavour Fragr. J..

[17] Gonçalves N.D., Grosso C.R.F., Rabelo R.S., Hubinger M.D., Prata A.S. (2018). Comparison of Microparticles Produced with Combinations of Gelatin, Chitosan and Gum Arabic. Carbohydr. Polym..

[18] Jerković I., Kuś P.M., Carbonell-Barrachina Á.A. (2019). Volatile Organic Compounds as Artefacts Derived from Natural Phytochemicals Sourced Form Plants and Honey. Phytochem. Rev..

[19] Jakab E., Blazsó M., Barta-Rajnai E., Babinszki B., Sebestyén Z., Czégény Z., Nicol J., Clayton P., McAdam K., Liu C. (2018). Thermo-Oxidative Decomposition of Lime, Bergamot and Cardamom Essential Oils. J. Anal. Appl. Pyrolysis.

[20] PubChem (National Library of Medicine). https://pubchem.ncbi.nlm.nih.gov/.

[21] ChemSpider (Royal Society of Chemistry). https://www.chemspider.com/.

[22] The Good Scent Company Informational System. https://www.thegoodscentscompany.com/.

[23] Peters H.J.W., van Bommel E.M., Fokkens J.G. (1992). Effect of Gelatin Properties in Complex Coacervation Processes. Drug Dev. Ind. Pharm..

[24] Comunian T.A., Thomazini M., Alves A.J.G., Junior F.E.d.M., Balieiro J.C.d.C., Favaro-Trindade C.S. (2013). Microencapsulation of Ascorbic Acid by Complex Coacervation: Protection and Controlled Release. Food Res. Int..

[25] Alvim I.D., Grosso C.R.F. (2010). Microparticles Obtained by Complex Coacervation: Influence of the Type of Reticulation and the Drying Process on the Release of the Core Material. Ciência e Tecnol. Aliment..

[26] Rocha-Selmi G.A., Bozza F.T., Thomazini M., Bolini H.M.A., Fávaro-Trindade C.S. (2013). Microencapsulation of Aspartame by Double Emulsion Followed by Complex Coacervation to Provide Protection and Prolong Sweetness. Food Chem..

[27] Ocak B. (2012). Complex Coacervation of Collagen Hydrolysate Extracted from Leather Solid Wastes and Chitosan for Controlled Release of Lavender Oil. J. Environ. Manag..

[28] Burhan A.M., Abdel-Hamid S.M., Soliman M.E., Sammour O.A. (2019). Optimisation of the Microencapsulation of Lavender Oil by Spray Drying. J. Microencapsul..

[29] Rungwasantisuk A., Raibhu S. (2020). Application of Encapsulating Lavender Essential Oil in Gelatin/Gum-Arabic Complex Coacervate and Varnish Screen-Printing in Making Fragrant Gift-Wrapping Paper. Prog. Org. Coat..

[30] Zhang R., Huang L., Xiong X., Qian M.C., Ji H. (2020). Preparation and Release Mechanism of Lavender Oil Microcapsules with Different Combinations of Coating Materials. Flavour Fragr. J..

[31] Musa H.H., Ahmed A.A., Musa T.H. (2019). Chemistry, Biological, and Pharmacological Properties of Gum Arabic. Bioactive Molecules in Food.

[32] Rousi Z., Malhiac C., Fatouros D.G., Paraskevopoulou A. (2019). Complex Coacervates Formation between Gelatin and Gum Arabic with Different Arabinogalactan Protein Fraction Content and Their Characterization. Food Hydrocoll..

[33] Napiórkowska A., Szpicer A., Wojtasik-Kalinowska I., Perez M.D.T., González H.D., Kurek M.A. (2023). Microencapsulation of Juniper and Black Pepper Essential Oil Using the Coacervation Method and Its Properties after Freeze-Drying. Foods.

[34] Larkin P.J. (2018). IR and Raman Spectra–Structure Correlations: Characteristic Group Frequencies. Infrared and Raman Spectroscopy.

[35] Stuart B.H. (2004). Infrared Spectroscopy: Fundamentals and Applications. Analytical Techniques in the Sciences.

[36] Shaddel R., Hesari J., Azadmard-Damirchi S., Hamishehkar H., Fathi-Achachlouei B., Huang Q. (2018). Use of Gelatin and Gum Arabic for Encapsulation of Black Raspberry Anthocyanins by Complex Coacervation. Int. J. Biol. Macromol..

[37] Milano F., Masi A., Madaghiele M., Sannino A., Salvatore L., Gallo N. (2023). Current Trends in Gelatin-Based Drug Delivery Systems. Pharmaceutics.

[38] Yang W., Gong Y., Wang Y., Wu C., Zhang X., Li J., Wu D. (2024). Design of Gum Arabic/Gelatin Composite Microcapsules and Their Cosmetic Applications in Encapsulating Tea Tree Essential Oil. RSC Adv..

[39] Santos M.G., Bozza F.T., Thomazini M., Favaro-Trindade C.S. (2015). Microencapsulation of Xylitol by Double Emulsion Followed by Complex Coacervation. Food Chem..

